# Ancient DNA of narrow-headed vole reveal common features of the Late Pleistocene population dynamics in cold-adapted small mammals

**DOI:** 10.1098/rspb.2022.2238

**Published:** 2023-02-22

**Authors:** Mateusz Baca, Danijela Popović, Alexander K. Agadzhanyan, Katarzyna Baca, Nicholas J. Conard, Helen Fewlass, Thomas Filek, Michał Golubiński, Ivan Horáček, Monika V. Knul, Magdalena Krajcarz, Maria Krokhaleva, Loïc Lebreton, Anna Lemanik, Lutz C. Maul, Doris Nagel, Pierre Noiret, Jérome Primault, Leonid Rekovets, Sara E. Rhodes, Aurélien Royer, Natalia V. Serdyuk, Marie Soressi, John R. Stewart, Tatiana Strukova, Sahra Talamo, Jarosław Wilczyński, Adam Nadachowski

**Affiliations:** ^1^ Centre of New Technologies, University of Warsaw, Warsaw, Poland; ^2^ Borissiak Paleontological Institute, Russian Academy of Sciences, Moscow, Russia; ^3^ Department of Early Prehistory and Quaternary Ecology and; ^4^ Senckenberg Centre for Human Evolution and Palaeoenvironment, University of Tübingen, Tübingen, Germany; ^5^ Department of Human Evolution, Max Planck Institute for Evolutionary Anthropology, Leipzig, Germany; ^6^ Department of Palaeontology, University of Vienna, Vienna, Austria; ^7^ Department of Zoology, Charles University, Prague, Czechia; ^8^ Department of Archaeology, Anthropology and Geography, University of Winchester, Winchester, UK; ^9^ Institute of Archaeology, Nicolaus Copernicus University in Toruń, Toruń, Poland; ^10^ Institute of Plant and Animal Ecology, Ural Branch, Russian Academy of Sciences, Yekaterinburg, Russia; ^11^ Department of Human and Environment, (HNHP) UMR 7194MNHN-CNRS-UPVD, National Museum of Natural History, Paris, France; ^12^ Catalan Institute of Human Paleoecology and Social Evolution (IPHES-CERCA), Tarragona, Spain; ^13^ Department of History and Art History, Rovira i Virgili University, Tarragona, Spain; ^14^ Institute of Systematics and Evolution of Animals, Polish Academy of Sciences, Cracow, Poland; ^15^ Senckenberg Research Station of Quaternary Palaeontology, Weimar, Germany; ^16^ Research Group Prehistory, University of Liège, Liège, Belgium; ^17^ DRAC/SRA Poitou-Charentes, Ministry of Culture and Communications, Poitiers, France; ^18^ Wrocław University of Environmental and Life Sciences, Wrocław, Poland; ^19^ Interdisciplinary Center for Archaeology and Evolution of Human Behavior, University of Algavre, Faro, Portugal; ^20^ Biogéosciences, UMR 6282 CNRS, University of Burgundy, Dijon, France; ^21^ Faculty of Archaeology, Leiden University, Leiden, The Netherlands; ^22^ Faculty of Science and Technology, Bournemouth University, Poole, UK; ^23^ Department of Chemistry G. Ciamician, Alma Mater Studiorum, University of Bologna, Bologna, Italy

**Keywords:** paleoclimate, climate change, interstadials, habitat, mitochondrial DNA, Pleistocene

## Abstract

The narrow-headed vole, collared lemming and common vole were the most abundant small mammal species across the Eurasian Late Pleistocene steppe-tundra environment. Previous ancient DNA studies of the collared lemming and common vole have revealed dynamic population histories shaped by climatic fluctuations. To investigate the extent to which species with similar adaptations share common evolutionary histories, we generated a dataset comprised the mitochondrial genomes of 139 ancient and 6 modern narrow-headed voles from several sites across Europe and northwestern Asia covering approximately the last 100 thousand years (kyr). We inferred Bayesian time-aware phylogenies using 11 radiocarbon-dated samples to calibrate the molecular clock. Divergence of the main mtDNA lineages across the three species occurred during marine isotope stages (MIS) 7 and MIS 5, suggesting a common response of species adapted to open habitat during interglacials. We identified several time-structured mtDNA lineages in European narrow-headed vole, suggesting lineage turnover. The timing of some of these turnovers was synchronous across the three species, allowing us to identify the main drivers of the Late Pleistocene dynamics of steppe- and cold-adapted species.

## 1. Introduction

There is growing evidence that the Late Pleistocene climatic and environmental oscillations have been a major factor shaping the evolutionary histories of many mammalian species [1–3]. Climate and environmental change in the Northern Hemisphere have led to dramatic demographic and genetic changes, either causing the extinction of whole genetic lineages and species or engendering a significant change in their ranges over a short period of time and large areas [4–6]. Most of the genetic studies have focused on megafauna species, such as mammoths, bears, horses and others [2,4,7]. However, less is known about the Late Pleistocene evolutionary histories of small mammals. The notable exceptions are the ancient DNA investigations of the collared lemming (*Dicrostonyx torquatus*) [8–11] and the common vole (*Microtus arvalis*) [12,13]. These studies revealed that both species experienced several mtDNA lineage replacements across most of the European continent during marine isotope stage (MIS) 3 and MIS 2 [8,9,13].

The narrow-headed vole (genus *Stenocranius* Kashchenko, 1901) is distinguished from all other voles by a high and narrow brain capsule. *Stenocranius* was originally proposed as a subgenus of *Microtus* Schrank, 1798 but in recent years phylogenetic reconstructions supported the sister relationship of *Stenocranius* and *Lasiopodomys* Lataste, 1887 [14] and *Stenocranius* has been accepted as a subgenus of the latter. However, the fossil record suggests an early split (*ca* 1.2 Ma) of *Stenocranius* from the *Allophaiomys* stock [15] and recent mitogenomic analyses showed divergence of *Stenocranius* and *Lasiopodomys* similar to the divergence between many genera within Arvicolini [16]. We, therefore, follow Kryštufek & Shenbrot [17] and recognize a genus status for *Stenocranius.* Narrow-headed vole are presently only found in Asia, in the belt of arctic tundra and forest-tundra in the north and the steppes and forest-steppes at the centre of the continent. These animals inhabit open areas, such as tundra, steppes and meadows [17]. Their current distribution in Asia is considered the result of habitat fragmentation during the Holocene [18]. The range of *Stenocranius* was much larger when treeless steppe-tundra was the dominant biome during the Pleistocene and included most of Northern Eurasia. The westernmost part of their former range reached the British Isles [19] and southern France [20]. Recent genetic investigations have shown that the Pleistocene narrow-headed voles were a complex of three cryptic species, including *Stenocranius anglicus* (Hinton, 1910) in Europe [21] and *Stenocranius gregalis* (Pallas, 1779) and *Stenocranius raddei* (Poljakov, 1881) in Asia [22]. The divergence of these three lineages was estimated to have occurred between 250 and 200 kyr ago by adopting the substitution rate of mtDNA cytochrome *b* from other vole species [21]. These substitution rates were estimated using recent calibration points, either directly radiocarbon-dated specimens [23] or biogeographic events [24]. Despite this, the estimated divergence time of the European and Asiatic populations has challenged the widely accepted biogeographic hypothesis, assuming that all narrow-headed-voles retreated from Europe during the interglacial periods and suggested that they survived the Eemian interglacial (MIS 5e, *ca* 128–115 ka) in European refugia [21].

The narrow-headed vole, and other rodents adapted to open habitats and/or cold climates, such as the collared lemming and common vole, frequently made up to 90% of the Late Pleistocene small mammal assemblages across Western and Central Europe [20,25–27] and constitute a key element of steppe-tundra environments. Investigations into their evolutionary and phylogeographic history would enhance our understanding of species' responses to climate change and demonstrate to what extent species with similar environmental requirements share the same evolutionary pathways.

Here, we generated more than 100 mitogenomes from Late Pleistocene skeletal remains and reconstructed time-calibrated phylogenies to explore the putative effects of climatic and environmental changes on the dynamics of narrow-headed vole populations.

## 2. Material and methods

### (a) Samples

Specimens identified as *Stenocranius* sp. based on the morphology of the occlusal surface of the first lower molar were obtained from several sites across Europe and Asia. Before the genetic analyses the occlusal surface of the m1 tooth was photographed at the Institute of Systematic and Evolution of Animals, Polish Academy of Science.

### (b) DNA extraction, library preparation, enrichment and sequencing

Genetic analyses were performed in the Laboratory of Paleogenetics and Conservation Genetics, Centre of New Technologies, University of Warsaw. To minimize the probability of contamination, all pre-polymerase chain reaction (PCR) library preparation steps were carried out in a dedicated ancient DNA laboratory separated from the post-PCR area. This laboratory has positive air pressure, and is UV irradiated after each use; researchers wore full protective suits.

Vole teeth were flushed twice with ultra-pure water in a sterile tube and crushed with a pipette tip. The DNA was extracted using a silica bead-based protocol optimized for retrieving ultrashort DNA molecules [28,29]. A negative control was processed with each batch of 15 samples to monitor possible contamination. One-third of each DNA extract was converted into double-indexed and either double- or single-stranded DNA sequencing libraries following the protocols proposed by [30] or [31], respectively. In each case, the double indexing scheme was introduced during 19 cycles of indexing PCR, using either AmpliTaq Gold or AccuPrime *Pfx* DNA Polymerase.

The libraries were enriched for vole mtDNA using a custom DNA bait produced from a range of vole species and following a protocol proposed by [32]. Up to five libraries were pooled in a single enrichment reaction which was carried out twice at 65°C for 22–24 h. After each round, the library pools were amplified in triplicate for 10–15 cycles using Herculase II Fusion Polymerase. The enriched library pools were combined, quantified using Qubit 4 and sequenced on an Illumina NextSeq550 platform (MID output, 2 × 75 bp kit). Additional details are provided in the electronic supplementary material, text S1.1–1.4 and table S1*.*

### (c) Processing the sequencing data

Sequencing reads were demultiplexed using bcl2fastq. Overlapping reads were collapsed and adaptor and quality trimmed using AdapterRemoval v. 2 [33]. The reads were mapped to the *S. gregalis* mtDNA genome (MN199170.1) using the *bwa mem* algorithm with the default settings. As the mapping efficiency was low due to high sequence divergence between extant Asiatic and ancient European specimens, we constructed two additional mtDNA references using the most complete sequences of European *S. anglicus.* In some cases, we performed competitive mapping using concatenated vole and human mtDNA genomes as a reference to remove human DNA contamination [34]. Duplicates and low mapping quality (mapq < 30) reads were removed using *samtools*. Variants and consensus sequences were called using the *bcftools mpileup* and *ivar call* commands. Positions with coverage lower than 3 were masked, and bases supported by less than 75% of the reads were coded with the appropriate IUPAC symbol. MapDamage v. 2.08 [35] was used to assess the damage patterns and length distribution of the DNA molecules. Additional details are provided in electronic supplementary material, text S1.5.

### (d) Radiocarbon dating

We attempted to radiocarbon date 12 specimens ranging in weight from 21.5 to 102 mg. Collagen was extracted and quality was assessed in the Department of Human Evolution at the Max Planck Institute for Evolutionary Anthropology (Leipzig, Germany) following the protocol for less than 100 mg bone samples described in [36]. Samples were combusted and converted to graphite using the AGE system [37] and dated using the MICADAS system [38] Radiocarbon dates were calibrated in OxCal v. 4.4 [39] using the IntCal20 [40] calibration curve. See electronic supplementary material, text S1.6 for further details.

### (e) Data analysis

The mtDNA sequences of the ancient and modern narrow-headed vole were aligned using MAFFT v. 7.407 [41]. The best-fitting partitioning scheme was found using PartitionFinder v. 2 [42]. Phylogenetic analyses were performed in BEAST v. 1.10.4 [43]. We used the Bayesian evaluation of temporal signal (BETS) [44] and the date-randomisation test [45] on the dataset consisting of all dated and modern sequences (*n* = 17) to determine whether there was enough temporal resolution to calibrate the molecular clock. Next, we performed a leave-one-out analysis on the directly dated specimens to check the accuracy of the molecular age estimate. In this analysis, we estimated the age of each directly dated specimen using all of the other dated specimens to calibrate the molecular clock. Then, we estimated the age of each undated specimen separately using directly dated specimens to calibrate the molecular clock. In each of the 128 separate BEAST analyses, we set the gamma prior (shape = 2; scale = 50 000) on the age of the undated specimen and increased the operator weight of the age estimate to 5. Finally, we ran a joint analysis using all of the sequences and setting the lognormal priors on the ages of undated specimens. The priors were set to match the posteriors of the age estimates from the individual analyses. See electronic supplementary material, text S1.7–1.8 and tables S2–S6 for more details.

To include the full previously recognized mtDNA diversity of Asiatic narrow-headed voles, we also reconstructed the phylogeny using an 882 bp fragment of mtDNA cytochrome *b*. This dataset was comprised 311 sequences (139 from ancient and 172 from extant specimens). See electronic supplementary material, text S1.9 and tables S7–S9, S11, for more details.

## 3. Results

**Figure 1.**
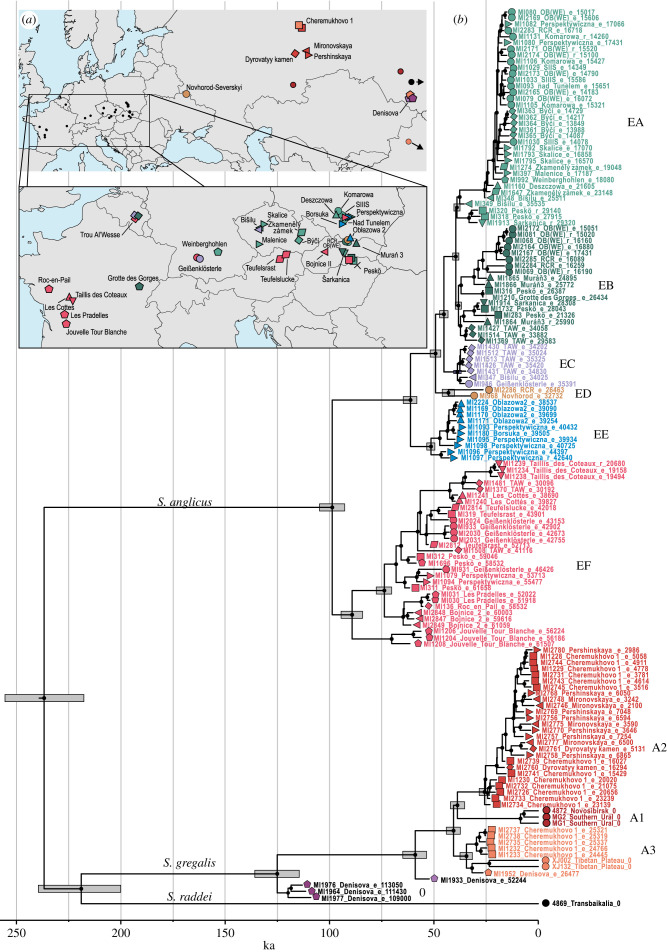
Phylogeny of last glacial period narrow-headed voles. (*a*) Sampling locations for the vole specimens. Colours and symbols correspond to mtDNA lineages and sampling sites. (*b*) Tip-calibrated phylogeny of narrow-headed voles based on the mtDNA genomes. Dots at nodes indicate posterior probability greater than 0.95. Grey bars represent 95% HPD intervals of the estimated divergence times. Tips are annotated with sample ID, sampling site and median of estimated (‘e’ prefix) or radiocarbon age (‘r’ prefix). Sites abbreviations: TAW, Trou Al'Wesse; RCR, Rock overhang in Cisowa Rock; SIIIS, Shelter in Smoleń III; OB(WE), Obłazowa (Western Entrance).

We recovered near complete (greater than 70% sites with a minimum coverage of 3 reads) mtDNA genomes from 139 specimens from 35 sites across Europe and western Asia. Together with the sequences of extant voles available from repositories, our dataset consisted of 145 mitogenomic sequences (electronic supplementary material, tables S8 and S9; figure 1). All ancient specimens yielded short inserts and an elevated level of deamination at the ends of the DNA molecules typical of ancient DNA (electronic supplementary material, table S8). Of the 12 specimens large enough for radiocarbon dating, 11 yielded collagen of sufficient quantity and quality for AMS dating (electronic supplementary material, table S10). The mtDNA sequences of these 11 specimens and the six extant specimens constituted a directly dated dataset used to estimate the ages of the remaining specimens. BETS analysis and the date-randomization test indicated that, the ‘dated’ dataset had a sufficient temporal signal to calibrate the molecular clock (electronic supplementary material, text S1.7, figure S1 and table S3). In the leave-one-out analysis, the 95% highest posterior density (HPD) intervals of the ages estimated for 10 of 11 specimens overlapped with their radiocarbon age. In most cases, the estimates were accurate, with the difference between the medians of the estimated ages and the radiocarbon ages ranged between 0.4% and 35% (median difference, 4.6%) of the latter (electronic supplementary material, figure S2). As a result, the age of the undated specimens estimated using the molecular approach was usually highly consistent with the stratigraphic position of the specimens and with the age estimates obtained for other species from the same sites and layers. The exception was some of the European specimens from the Early Holocene layers whose ages were slightly but systematically overestimated compared to their stratigraphic position (electronic supplementary material, table S8). However, the youngest estimated ages (*ca* 14 ka; electronic supplementary material, table S1), corresponded well with the beginning of the Bølling–Allerød interstadial (14.7–12.9 ka), suggesting earlier extinction of the narrow-headed vole from central Europe. We also note that in the final joint phylogenetic analysis of the 145 mitogenomes the MCMC chains for age estimates of six specimens did not converge (effective sample size [ESS] < 200) in both replicates. However, the age parameters were reliable (ESS > 200) in individual analyses of all of these samples, and yielded values very similar to those obtained in the final joint analysis (electronic supplementary material, text S1.8 and table S6); thus, we consider these estimates to be reliable.

### (a) Diversification of the narrow-headed vole mtDNA lineages

The Bayesian phylogeny based on mitochondrial genomes recovered three divergent lineages corresponding to cryptic species identified previously, including *S. anglicus* in Western and Central Europe, and *S. gregalis* and *S. raddei* in Asia [21,22,46]. The three lineages diverged at similar times during the MIS 7 interstadial (243–191 ka). The divergence of the *S. anglicus* and *S. gregalis/S. raddei* lineages was estimated to be 233 ka (95% HPD: 253–213 ka) and that of the *S. gregalis* and *S. raddei* lineages was 216 ka (95% HPD: 236–193 ka; figure 1).

The *S. anglicus* lineage further diversified about 98 ka (95% HPD: 104–92 ka) during MIS 5c (∼GS-23; *ca* 104–88 ka). Our mitogenomic tree does not reflect the complete diversity of Asiatic *S. gregalis,* but the tree based on the mtDNA cytochrome *b* and including all extant lineages of this species had a similar tMRCA of 95 ka (95% HPD: 112–80 ka; electronic supplementary material, text S1.9, tables S11 and S12, figure S3). The three specimens from layers 14 and 12.3 of the Denisova cave with ages estimated using the molecular approach to be between 116 and 107 ka fell outside the mtDNA diversity of all of the last glacial period (*ca* 113–11.7 ka) *S. gregalis* specimens. The estimated age of the specimen from layer 12.3 was in very good agreement with the dating of this layer (146–121 ka), while the estimated ages of the two specimens from layer 14 were slightly younger than the estimated time of deposition of this layer (202–163 ka) [47].

### (b) Temporal population structure and dynamics of the Asiatic narrow-headed vole

The geographical scope of our *S. gregalis* mitogenomic dataset was limited and included four sites in the Northern and Middle Urals and one in Altai. However, the phylogeny based on mtDNA cytochrome *b,* which included 172 modern specimens, was consistent with previous studies and consisted of three main lineages (A–C). Lineage A was further subdivided into six subclades (A1–A6) (electronic supplementary material, figure S3). The diversification of these sublineages was estimated to occur between 51 and 28 ka. A single specimen from layer 11.3 in Denisova cave was placed in a sister position to the A lineage. Its age was estimated to be about 52 (95% HPD: 58–46) ka only slightly younger than the estimated deposition period of this layer (100–61 ka). Among the ancient specimens, those older than *ca* 23 ka belonged to lineage A3, which is currently limited to Altai. Lineage A3 was replaced by A2 *ca* 23 ka in the Northern and Middle Urals and the latter persisted there until the Middle Holocene. Currently, A2 occurs mostly to the north, on the Yamal Peninsula (electronic supplementary material, figure S4). This finding supports previous reports suggesting that the range of Asiatic narrow-headed voles became fragmented during the Holocene [18].

### (c) Temporal population structure and dynamics of the European narrow-headed vole

We observed six *S. anglicus* mtDNA lineages in Europe, which we labelled EA–EF (figure 1). The EF lineage was distributed across most of Europe since at least 60 ka and until *ca* 44–42 ka (figures 1 and 3). Then, its range decreased and eventually, it was limited to the territory of what is now southwestern France where it persisted until at least 17 ka. Lineage EE had a limited geographical range, as it was only found at three sites from what is modern-day Poland (Obłazowa 2, Perspektywiczna layer 7c and Borsuka layer VI) among specimens with ages estimated to be between 44.4 and 38.5 ka. Lineage ED was attributed to only two specimens from two sites in what is modern-day Poland and Ukraine (Novgorod Severskyi and a rock overhang at Cisowa Rock). In both cases, the estimated ages were significantly older than suggested by the site's stratigraphy. Lineage EC was found in western and central Europe (Trou Al'Wesse, Geißenklösterle and Bišilu) in specimens with ages estimated to be between 35.4 and 34.2 ka. Lineages EB and EA diverged about 40 ka. The oldest specimens from these two lineages had similar ages and came from western (Trou Al'Wesse, Belgium, *ca* 34 ka) and central Europe (Bišilu, Czechia, *ca* 35 ka), respectively. Lineage EB appeared in central Europe 28 ka when its range started to overlap with the EA lineage, which remained restricted to central Europe.

## 4. Discussion

### (a) Impact of the interglacial periods on cold-adapted small mammals

Our results based on ancient mitogenomic data indicate that the divergence of the three main narrow-headed vole lineages occurred during the MIS 7 interstadial (243–191 ka). These divergence estimates are much younger than those of *S. gregalis* and *S. raddei* based on the fossil calibration [16,46]. This is likely to be due to time dependence of molecular rate estimates [48]. There is compelling evidence that the estimates of evolutionary rates are negatively correlated with the age of calibration points and the use of fossil calibration may lead to overestimation of recent divergence times [49].

**Figure 2.**
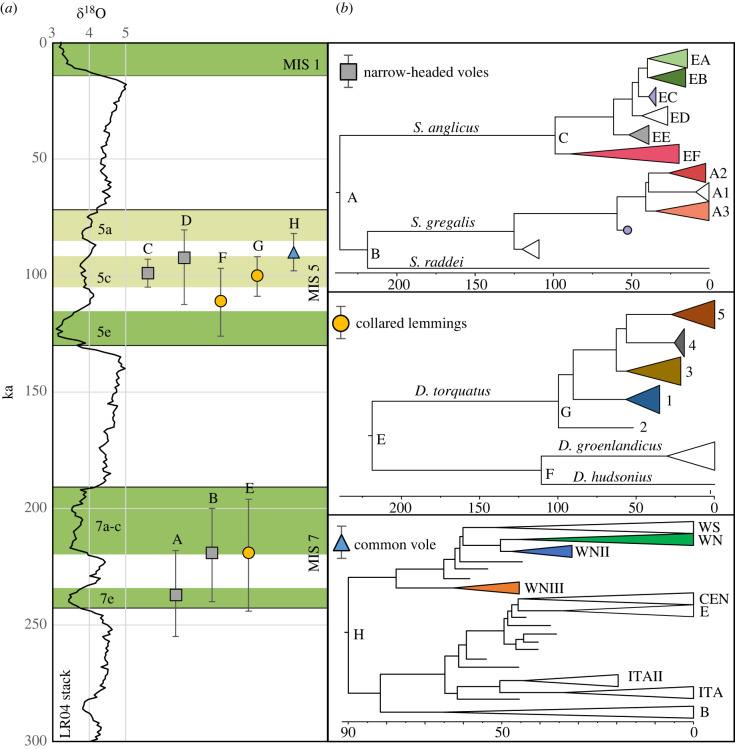
Divergence times of the main lineages within the three species groups. (*a*) Divergence times (medians and 95% HPDs) plotted against the benthic foraminifera *δ*^18^O record (LR04 stack; [50]). Interglacial and interstadial periods mentioned in the text are marked with green strips. (*b*) Schematic mitochondrial phylogenies of the narrow-headed voles, collared lemmings and common vole. Letters at the nodes correspond to the divergence times in (a). The age of diversification of modern *S. gregalis* lineages (D) was taken from the mtDNA cytochrome *b* phylogeny (see electronic supplementary material, figure S3 and text S1.9). Symbols at tips indicate the main mtDNA lineages within each species. Colours indicate mtDNA lineages presented in figure 3. Data for collared lemming are from [11] and for common vole from [12].

Our estimates are very similar to the estimated divergence of the two main lineages of collared lemming, Eurasian (*D. torquatus*) and North American (*D. groenlandicus/D. hudsonius*), based on ancient mitogenomes (figure 2). The available palynological records from MIS 7 suggest the expansion of mixed coniferous-broadleaved forest across Europe, from southern France [51] to the Eastern European Plain [52] possibly forcing narrow-headed voles to retreat to refugia in Europe and Asia, although the location of these areas is unknown. The sea level during the warm phases of MIS 7 (MIS7a–c, e) was only marginally lower than it is today [53], resulting in flooding of the Bering Strait and the separation of the Eurasian and American collared lemming populations.

The estimated age of diversification of the main mtDNA lineages found in the European and Asiatic narrow-headed voles from the last glacial period was similar to the estimated diversification of the main lineages within common voles [12] and Eurasian collared lemmings [11] that occurred during MIS 5c (Brørup interstadial; ∼GI 23) (figure 2).

Temperate deciduous vegetation developed across Europe during Brørup (MIS 5c) [54,55]. The presence of *Quercus* followed by *Carpinus* woodlands was evidenced in France. A high percentage of *Betula* pollen was detected in Northern Germany, followed by *Pinus* with admixtures of *Picea* and *Larix*. In central Europe, the development of *Betula* forests followed by *Betula–Pinus* and *Pinus–Picea–Larix* phases has been observed in the palynological records. The development of dense forests over vast areas of Europe may have led to fragmentation of the populations adapted to open, steppe habitats, leading to the divergence and formation of new lineages.

Taken together, these observations suggest a common response of species adapted to open habitats in interglacial environments. Similar contraction during the Eemian interglacial period followed by expansion during the last glacial period was recently identified in a range of steppe insect and plant species [56].

As mentioned in the Introduction, the long-held notion that narrow-headed voles withdrew completely from Europe during the Eemian (and penultimate interglacial periods) cannot be maintained due to the high divergence of the European narrow-headed voles from Asian populations shown previously [21]. Narrow-headed voles can theoretically survive unfavourable interglacial conditions in mountain/upland areas or in the far north, where open and relatively humid environments persisted. However, the fossil record does not provide evidence for the species in Europe at this time, despite the examination of over 90 sites of Eemian age in Europe [57]. *S*. cf *anglicus* was only found in two sites in Hungary and one in Poland although Eemian dates could not be confirmed. By contrast, *S. anglicus* appeared in great numbers at the very beginning of the last glaciation during MIS 5d–a in various parts of Europe, such as the Rhone Valley and the Massif Central in France [58], the Ach Valley in Germany [59] and the Kraków-Częstochowa Upland of Poland [60]. This may suggest that the narrow-headed vole survived the Eemian interglacial in several refugia, and therefore their rapid re-colonization of Europe was possible as soon as the climatic cooling offered a suitable environment for its expansion. This would confirm the proposed idea of cryptic refugia for continental adapted species in more oceanic areas in the past [61].

### (b) Narrow-headed vole population dynamics during the last glacial period

**Figure 3.**
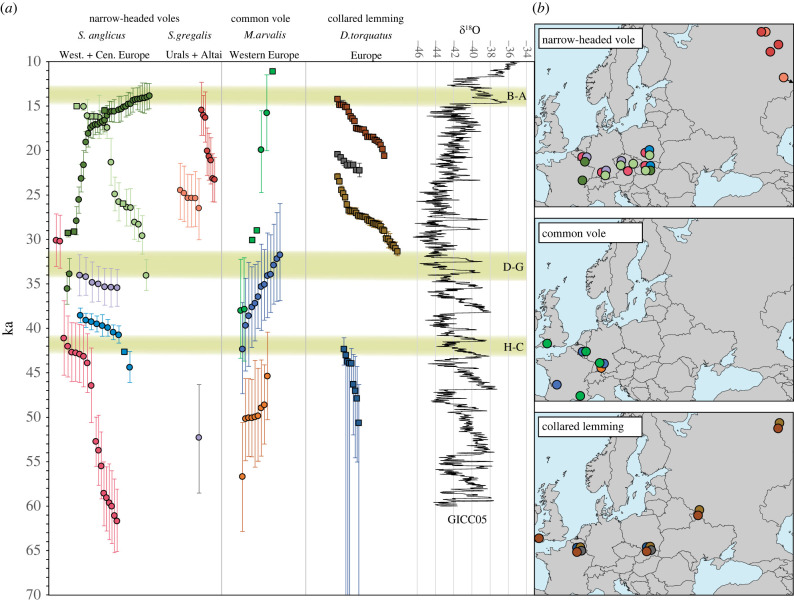
Temporal distribution of the mtDNA lineages across four steppe- and cold-adapted rodent species (*a*). Circles represent medians and whiskers are the 95% HPDs of the specimen ages estimated using the tip-dating approach; squares represent medians of the calibrated radiocarbon ages with 95.4% probability ranges. In the case of narrow-headed vole data from the present-day western France, where lineage EF was present continuously, was omitted. The phylogenetic relationships between the presented mtDNA lineages are shown in figure 2*b*. Data for the collared lemming are from [8,9] and for the common vole from [12]. The oxygen isotope record (*δ*^18^O V-SMOW) from the Greenland Ice cores (GICC05) [62] is given on the right side. Green stripes denote the main interstadials identified from the palynological records within the last 50 kyr (H-C: Hengelo-Charbon; D-G: Denekamp-Grand Bois; B-A: Bølling–Allerød). (*b*) Geographical ranges of the mtDNA lineages presented in (*a*). Nearby localities were merged for better visibility.

Our phylogenetic reconstruction suggests a highly dynamic history of the European narrow-headed vole through the last glacial period. We identified six mtDNA lineages in western and central Europe. Most of them appeared consecutively within the limits of our resolution, suggesting genetic turnover across the region. Some of the lineages (e.g. EE) had limited geographical distributions. This is likely to be an effect of sampling bias and the short temporal range of these lineages. Nevertheless, the full geographical scope of the recorded turnovers remains to be described. Most of the turnover in the mtDNA lineages of the European narrow-headed vole, the collared lemming and the common vole occurred at the end of MIS 3 between *ca* 45 and 30 ka (figure 3). The limited data available for the Asiatic *S. gregalis,* with divergence times for the A1–A6 lineages estimated to be between 51 and 28 ka, also suggest a major reorganization of genetic diversity during this period. This is broadly consistent with the cluster of megafaunal extinctions and mtDNA lineage turnovers reported previously at the end of MIS 3, between 40 and 26 ka [5,63–65], including that of the Neanderthals [66]. The main cause of the megafauna turnovers has been argued to be the abrupt warming at the onset of the Greenland Interstadials (GI), particularly around GI7–GI5. The role of humans, exacerbating the impact of climate change, has also been emphasized [5,65], as no turnovers were detected before modern humans appeared in Europe *ca* 45 ka. The direct impact of Palaeolithic humans on the vole and lemming populations across Eurasia has been considered negligible. The concurrence of mtDNA lineage turnovers in small and large mammals at the end of MIS 3 suggest that the climate and environmental conditions may have been the sole drivers of the observed population dynamics.

The resolution of the molecular estimates of the ages of the specimens is limited, although a comparison of the temporal distribution of the mtDNA lineages of the three rodent species suggests that populations of all three rodent species were affected at similar times about 45–40 ka and about 32 ka (figure 3). These findings generally coincide with the timing of the interstadials identified in the European pollen records; Hengelo–Charbon dated to about 43–41 ka cal BP [54,67] and Denekamp–Grand Bois dated to about 36–33 ka cal BP [54,55]. The correlation of these interstadials with the Greenland ice core records is challenging due to the relatively wide date error ranges and that Hengelo–Charbon is usually associated with Greenland Interstadial 11 (GI-11; *ca* 43.3–42.2 ka) or GI-10 (*ca* 41.5–40.8 ka) and Denekamp–Grand Bois is associated with GI-8 (38.2–36.6 ka) [54,68]. Both interstadials were characterized by the emergence of forests, although especially during the Hengelo–Charbon period the landscape remained relatively open albeit the steppe-tundra biome significantly altered [54,55]. Another factor that may have contributed to the observed dynamics was a period of weakend magnetic field before the Laschamps geomagnetic inversion. It has been suggested as a cause of major environmental rearrangements *ca* 42 ka. A similar paleomagnetic anomaly, the Mono Lake event, that occurred about 34 ka was hypothesized to cause similar effects [69]. Nevertheless, the main cause of these events, the synchronous faunal turnovers and local extinctions observed across species of different sizes and occupying different trophic levels, support a major ecosystem rearrangement at that time.

Following the theory of a major impact of rapid environmental change on animal populations, few megafaunal turnovers were recorded around the Last Glacial Maximum (LGM; *ca* 23–19 ka), when the climate was harsh but stable compared to the end of MIS 3 [5]. By contrast, studies on collared lemmings have previously shown two mtDNA lineage turnovers during the LGM [8,9]. Our data suggest another turnover in the *S. gregalis* populations from the Northern and Middle Urals, estimated to be about 24–23 ka. No such turnover was visible in European populations, but we noted that all specimens younger than 21 ka coalesced around 24 ka, suggesting a reduction in the population followed by expansion around this time. The causes of these turnovers remain unclear. However, there are reports, confirmed by direct radiocarbon dating, of species described by some as temperate such as brown bears (*Ursus arctos*) at medium and high latitudes in Europe during the LGM [2,70]. It has been hypothesized that a steppe-tundra environment may have been replaced by more temperate habitat for short periods [2]. Some climate variability between 27 and 21 ka cal BP, which was not apparent in the Greenland *δ*^18^O records, was detected in the Nussloch loess sequence, indicating several short phases of milder climate [68]. The accumulation of radiocarbon dates from organic material reflecting a warmer period about 23 ka was also detected in metanalysis of radiocarbon dates from Polish sites [71]. This may explain the published results of brown bear and collared lemming as well as those for the narrow-headed vole reported here.

Interestingly, the highly dynamic history of the narrow-headed vole populations observed in central and part of western Europe did not extend to what is now western France, where lineage EF was continuously present from at least 61 to 19 ka and plausibly until the extirpation of narrow-headed voles from Europe at the beginning of Late Glacial, and/or the Pleistocene–Holocene transition. This observation suggests that during MIS 3 and MIS 2 the environmental conditions in this region were more stable and/or favourable for narrow-headed voles than in other parts of Europe. Unfortunately, no genetic data of other small mammalian species are available from the region to determine whether the observed population continuity was a common phenomenon across open habitat and/or cold-adapted species. A recent reconstruction, based on the small mammal fossil record, revealed the general stability of the rodent communities between 50 and 19 ka in the region [20], although such reconstructions have limited power to detect rapid population turnover.

## Data Availability

The consensus mtDNA sequences generated in this study have been deposited in GenBank under Accession numbers OM809882–OM810020. The mtDNA sequence alignments used to reconstruct the mitogenome and cytochrome phylogenies have been deposited in Dryad Digital Repository and can be accessed at https://doi.org/10.5061/dryad.kh1893295 [72]. The mitochondrial alignments generated in this study have been deposited in the European Nucleotide Archive under project number PRJEB56307. The data are provided in electronic supplementary material [73].
